# An Optical Frequency Domain Angle Measurement Method Based on Second Harmonic Generation

**DOI:** 10.3390/s21020670

**Published:** 2021-01-19

**Authors:** Wijayanti Dwi Astuti, Hiraku Matsukuma, Masaru Nakao, Kuangyi Li, Yuki Shimizu, Wei Gao

**Affiliations:** 1 Precision Nanometrology Laboratory, Department of Finemechanics, Tohoku University, Sendai 980-8579, Japan; wijayanti@nano.mech.tohoku.ac.jp (W.D.A.); mnakao@nano.mech.tohoku.ac.jp (M.N.); likuangyi@nano.mech.tohoku.ac.jp (K.L.); yuki.shimizu@nano.mech.tohoku.ac.jp (Y.S.); gaowei@nano.mech.tohoku.ac.jp (W.G.); 2 Department of Electrical Engineering and Informatics, Vocational School, Universitas Gadjah Mada, Yogyakarta 55281, Indonesia

**Keywords:** mode-locked laser, angle measurement, second harmonic generation, frequency-domain angle measurement, parabolic mirror

## Abstract

This paper proposes a new optical angle measurement method in the optical frequency domain based on second harmonic generation with a mode-locked femtosecond laser source by making use of the unique characteristic of the high peak power and wide spectral range of the femtosecond laser pulses. To get a wide measurable range of angle measurement, a theoretical calculation for several nonlinear optical crystals is performed. As a result, LiNbO_3_ crystal is employed in the proposed method. In the experiment, the validity of the use of a parabolic mirror is also demonstrated, where the chromatic aberration of the focusing beam caused the localization of second harmonic generation in our previous research. Moreover, an experimental demonstration is also carried out for the proposed angle measurement method. The measurable range of 10,000 arc-seconds is achieved.

## 1. Introduction

Angle is one of the fundamental quantities for precision metrology and manufacturing [1,2]. Optical measurements provide non-contact measurement solutions for these precision angular measurements and are preferred over contact measurement methods for measuring items with fragility, deformability, and ultra-precision. Optical rotary encoders are the most well-used optical sensors in industries for measurement of the rotational angle of the shaft of a rotary stage or a rotary motor [1]. In a rotary encoder, the circular scale graduations fabricated on a scale disk, which is mounted on the shaft axis of rotation, are read by an optical read head that is kept stationary. A rotary encoder can cover the full 360° range of the shaft rotation. On the other hand, in addition to the rotational angle measurement of a shaft over full revolutions, tilt angle measurement over a small range, such as the tilt error motions of a spindle in a machine tool, is also an important task [1]. Such a tilt error motion is typically smaller than ±1°. However, it is difficult to employ a rotary encoder for such a measurement since there is no shaft available for mounting the encoder disk. Other types of optical measurement methods, which can be referred to as the non-disk methods for clarity, including autocollimation [3,4,5], a method based on the internal-reflection effect [6], and interferometry-based angular measurement [7], in which the angular response of a reflector or a prism to an incident laser beam is utilized, have then been developed for tilt angle measurement over a small range. Differing from a rotary encoder in which a rotating shaft is required to mount the encoder disk and it being necessary to keep a small and constant gap between the disk and the read head, it is much easier and more flexible to arrange the laser beam and the target reflector/prism in the non-disk angle measurement methods. For this reason, such methods have also been applied to the geometrical measurement of an object. For instance, measurement of straightness [8,9,10,11], profile measurement of the surface [12,13], and measurement of multi-axis positions [14,15,16,17,18,19].

The conventional non-disk angle measurement methods use a continuous wave (CW) laser. The laser radiation widths are less than 0.1 nm, thus, they are called monochromatic lasers. On the other hand, recently, mode-locked lasers emitting femtosecond pulses have been used for precision measurements. The mode-locked laser is characterized by two features: broadband in wavelength and high intensity. A measurement using a CW laser is an incremental measurement. The disadvantages of incremental measurements are counting errors and the need for zero positionings in case of power loss. On the other hand, measurements by a mode-locked laser can provide absolute measurements due to its information regarding the wavelength [20,21,22,23,24]. We have developed an absolute angular measurement autocollimator by using the feature of broadband wavelength [20,21,22,23]. In this method, a mode-locked laser beam is injected into a diffraction grating placed on the measurement target, and the light of each dispersed wavelength is focused by a lens onto an optical fiber which is the detector. Since the wavelength of the focused light on an optical fiber varies with angular displacement, the angle can be determined by detecting the optical wavelength. Meanwhile, we have also developed an angular measurement method based on second harmonic generation (SHG) using high-intensity features [25]. In this method, a mode-locked laser is focused on a nonlinear optical crystal placed on the measurement target. The angle can be detected because the intensity of the generated second harmonic wave (SHW) varies depending on the angle between the optic axis of the nonlinear optical crystal and the laser propagation direction. Nonlinear optical phenomena, which are nonlinear with the optical intensity and are observed with pulsed lasers with high intensity, are one of the promising techniques that have been continuously developed since the first demonstration in 1961 [26,27,28,29]. This optical phenomenon has been used for various applications, such as the characterization of short and ultrashort pulsed lasers [30,31,32] and surface characterization [33,34], and is also used in image sensing techniques such as bioimaging [35,36,37,38].

An angular measurement method based on SHG [25] produces the power change of the converted SHW when a mode-locked laser is focused on a nonlinear optical crystal, in which the SHW has half the wavelength of the mode-locked laser and is detected by a photodiode. The efficiency depends on the angle between the optic axis of the nonlinear optical crystal and the laser propagation direction. The angle at which the SHW conversion efficiency is maximized is called the matching angle. In general, due to the dispersion relation of the refractive index, the matching angle depends on the wavelength. In a previous paper, beta-barium borate (BBO) was used as a nonlinear optical crystal, and the matching angle of BBO has a very small wavelength dependence over the spectral bandwidth from 1500 to 1620 nm of an Er-doped fiber laser [25]. Thus, the intensity of the SHW is not wavelength dependent but depends on the matching angle. Therefore, we developed an angular measurement method to obtain the angular displacement from the strength of the SHW. However, there were some drawbacks to this method. First, since the intensity of the SHW depends on the square of the fundamental wave (FW) intensity, the stabilization of the FW intensity increases the measurement uncertainty. Secondly, if the nonlinear optical crystal shifts from the point where the FW is focused, the intensity of the SHW decreases, and the measurement uncertainty is increased. Third, the measurable range is small, about 400 arc-seconds. Besides, the chromatic aberration of a lens employed in the proposed method reduces the wavelength bandwidth contributing to the SHW, since the localization of SHG occurs.

In this study, we propose an angular measurement method based on SHG in the optical frequency domain to overcome the above drawbacks. Using a nonlinear optical crystal with a large matching angle dispersion, the wavelength of the SHW depends on the angular displacement. In this method, the measurement uncertainty does not depend on the light intensity, and high measurement stability can be expected. Additionally, because the wavelength of the SHW depends on the angle of incidence of the FW, absolute angle measurement [20,21,22,23] is available. Therefore, we perform simulations on matching angles and wavelength dependence for various nonlinear optical crystals and obtain conditions with a wide measurable range of angles. The simulations also take into account refraction at the interface between the nonlinear optical crystal and the air. We present a feasibility study of angle measurements with a wide measurable range of angles based on calculations. In this study, we change from refractive optics to reflective optics to avoid the localization of SHG, which was a drawback of the focusing lens in the previous paper [25].

## 2. Theoretical Approach

### 2.1. The Difference of the Measurement Principle between the Previous and the Proposed Studies

**Figure 1:**
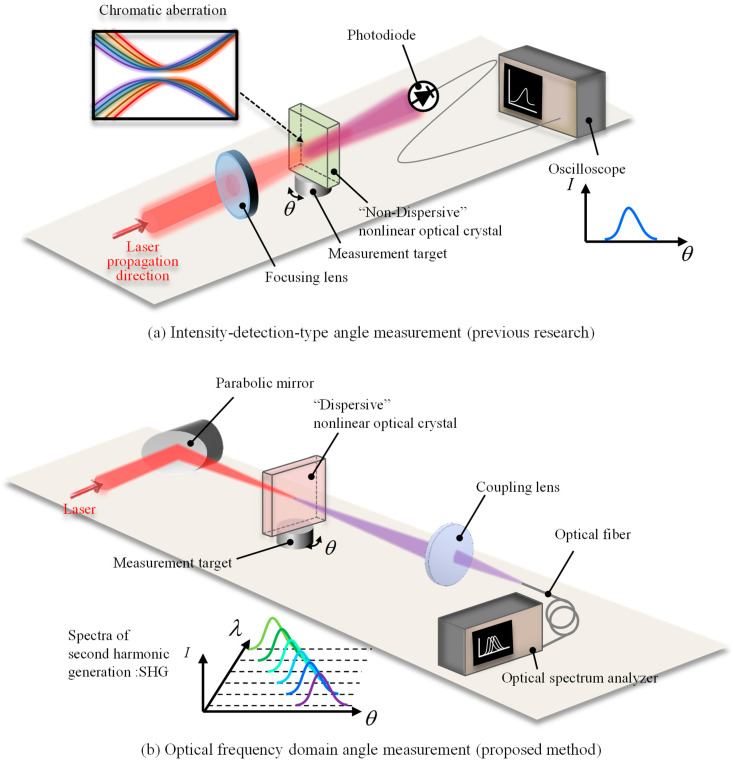
(**a**) A schematic of the previous research. (**b**) A schematic view of this research.

Before discussing the theoretical approach, an overview of the present study and the difference from the previous research is shown in Figure 1. In the previous research [25], the laser beam (FW light) is focused onto the nonlinear optical crystal (BBO), and converted to the SHW in the BBO. The power of the converted SHW is detected by a photodiode and recorded by an oscilloscope. The angular displacement of the measurement target is calculated from the obtained intensity. Meanwhile, in the proposed measurement system, the lens is replaced by a parabolic mirror to avoid chromatic aberration. The laser beam is focused by a parabolic mirror onto a nonlinear optical crystal and converted into an SHW, which is focused by a lens onto a multimode fiber, and spectra are recorded by an optical spectrum analyzer. From the obtained optical wavelengths, the angular displacement of the target is calculated.

### 2.2. Angle Dependence of Second Harmonic Generation

**Figure 2:**
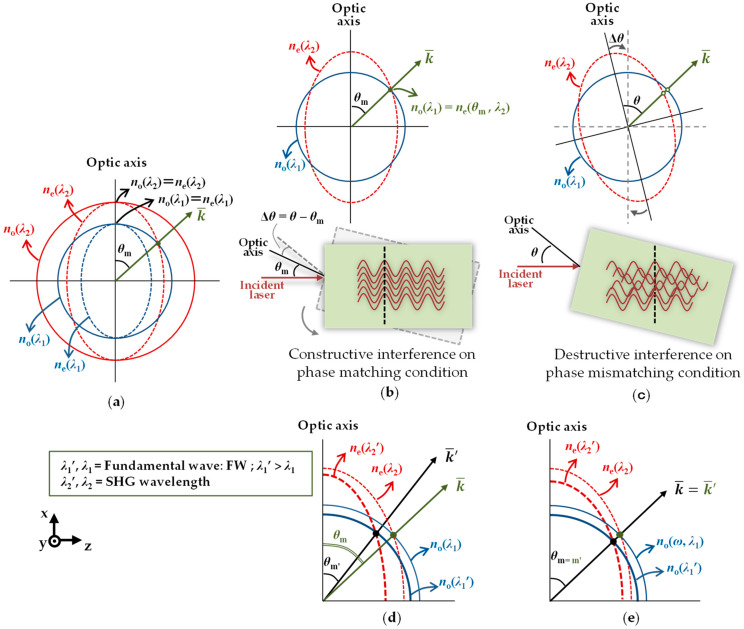
(**a**) Two-dimensional perspective of the refractive indices surface for type I negative uniaxial crystal; (**b**) phase matching condition; (**c**) phase mismatching condition; (**d**) the change on refractive indices’ surface followed by the change of phase-matching angle; (**e**) the change of refractive indices’ surface with the same phase-matching angle in a certain range of wavelengths.

Several kinds of anisotropic optical materials have birefringence. Birefringence is an optical property of a material whose refractive index depends on the polarization of light and the direction of propagation, that is to say, the refractive index of the ordinary beam (*n*_o_) does not depend on the propagation direction, whereas that of the extraordinary beam (*n*_e_) depends on the propagation direction. Consider the case of type I negative uniaxial crystals [28], as illustrated in Figure 2a, *n*_e_ is less than *n*_o_, therefore, FW propagates as an ordinary beam and SHG wave as an extraordinary beam. By adjusting *θ* to obtain the value of *n*_e_ for making the refractive index of FW equal to the refractive index of the SHG wave, i.e., *n*_o_(*λ*_1_) = *n*_e_(*θ*_m_, *λ*_2_), the wavenumber of both refractive indices (*k*) is equal, and the phase-matching condition is satisfied. At this angle *θ*_m_, the SHG output power gains the highest efficiency due to the constructive interference of SHW [28,39,40], as shown in Figure 2b. On the other hand, the case in which the phase-matching condition is not satisfied is shown in Figure 2c. Here, the interference between SHWs is destructive. Moreover, the refractive index also depends on the wavelength. A longer wavelength has a smaller refractive index in general. The phase matching angle depends on both *n*_o_(*λ*_1_) and *n*_e_(*θ*, *λ*_2_). As shown in Figure 2d, the BBO used in the previous research changes the matching angle *θ*_m_ slightly at around 1560 nm, when *n*_o_(*λ*_1_) and *n*_e_(*θ*, λ_2_) are changed. Therefore, it is ideal for intensity-based angle detection. On the other hand, in many crystals, changes in *n*_o_(*λ*_1_) and *n*_e_(*θ*, λ_2_) lead to changes in *θ*_m_, as shown in Figure 2e. For this reason, these crystals are preferred for the angle measurement method based on SHG in the frequency domain.

To understand the proposed angle measurement method qualitatively, the power of the SHW is discussed. For a certain wavelength, the power of the SHW, *P*_2_, varies with angular displacement, as shown in the following equation [28].

(1) $$P_{2}=\frac{8\pi^{2}{d_{\mathrm{eff}}}^{2}}{n_{o}{\left(\lambda_{1}\right)}^{2}n_{e}\left(\theta ,\lambda_{2}\right)\varepsilon_{0}c{\lambda_{1}}^{2}}\frac{L^{2}}{S}{P_{1}}^{2}{\sin c}^{2}\frac{\left|\Delta k(\theta )\right|L}{2}$$

where the effective nonlinear coefficient is denoted by *d*_eff_, while *λ*_1_ and *λ*_2_ are wavelengths of the FW and SHW, respectively, *ε*_0_ is the vacuum permittivity, *c* is the speed of light in a vacuum, *S* is the cross-sectional area of the focused beam, and *L* is the crystal length. Since the femtosecond laser is used as the laser source, the FW is estimated at its central wavelength, around 1560 nm. As shown in the equation, *P*_2_ is proportional to the square of the FW power *P*_1_ and is proportional to the square of the sinc function with respect to the angle between the FW propagation direction and the crystal axis. The sinc function is determined by the length of the nonlinear optical crystal and the phase mismatch (Δ*k*) as a function of incident angle to the optic axis of the nonlinear crystal (*θ*), as shown mathematically in the following equation.

(2) $${\sin c}^{2}\frac{\left|\Delta k(\theta )\right|L}{2}=\frac{\sin^{2}\left(\left|\Delta k(\theta )\right|L/2\right)}{{\left(\left|\Delta k(\theta )\right|L/2\right)}^{2}}$$

In this study, the phase mismatch can be obtained by considering the refractive index of the ordinary beam (*n*_o_) as the function of the wavelength of FW and the refractive index of the extraordinary beam (*n*_e_) as the function of the angle and wavelength of the SHW, which is mathematically expressed in the following equation [28].

(3) $$\Delta k\left(\theta \right)=\frac{4\pi }{\lambda_{1}}\left[n_{o}\left(\lambda_{1}\right)-n_{e}\left(\theta ,\lambda_{2}\right)\right]$$

For the transparent medium, the refractive index of the ordinary beam (*n*_o_) and the refractive index of the extraordinary beam (*N*_e_) of the FW can be calculated empirically by using the following equation.

(4) $$N_{e}\left(\lambda_{j}\right),n_{o}\left(\lambda_{j}\right)=\sqrt{A+\frac{B}{{\lambda_{j}}^{2}-C}-D{\lambda_{j}}^{2}}\;;\;j=1,2$$

Here, to simplify the notation, the condition of *n*_e_(90°, *λ*_j_) is denoted by *N*_e_(*λ*_j_) and the condition of *n*_e_(0°, *λ*_j_) is denoted by *n*_o_(*λ*_j_), while the medium-specific constants are denoted by the coefficients A, B, C, and D. The constants for beta-barium borate (BBO), lithium iodate (LiIO_3_), and lithium niobate (LiNbO_3_), as the parameters of the empirical calculation, are presented in Table 1.

Furthermore, to complete the calculation of Equation (3), the refractive index of the extraordinary beam as the function of *θ* and *λ*_2_ is expressed by the equation below [40].

(5) $$\frac{1}{{n_{o}}^{2}\left(\lambda_{1}\right)}=\frac{\cos^{2}\theta_{m}}{{n_{o}}^{2}\left(\lambda_{2}\right)}+\frac{\sin^{2}\theta_{m}}{{n_{e}}^{2}\left(\lambda_{2}\right)}$$

In the condition of *n*_o_(*λ*_1_) = *n*_e_(*θ*, *λ*_2_), Equation (5) can be transformed as follows.

(6) $$n_{e}\left(\theta ,\lambda_{2}\right)=\frac{1}{\sqrt{{\left[\sin\theta /N_{e}\left(\lambda_{2}\right)\right]}^{2}+{\left[\cos\theta /n_{o}\left(\lambda_{2}\right)\right]}^{2}}}$$

According to Equation (1), the highest SHG power can be achieved in the case of a sinc function equal to 1, that is to say, a phase mismatch condition equal to 0. To realize this condition, the incident angle of the laser beam must be set at a certain angle toward the optic axis of the nonlinear crystal, called the phase-matching angle. Thus, phase-matching conditions occur as Δ*k*(*θ*_m_) = 0 and *n*_o_(*λ*_1_) = *n*_e_(*θ*_m_, *λ*_2_). Since each nonlinear optical crystal has its specific phase-matching angle, it is necessary to estimate the matching angle of each crystal that satisfies with the following equations [39].

(7) $$\sin^{2}\theta_{m}=\frac{{n_{o}}^{-2}\left(\lambda_{1}\right)-{n_{o}}^{-2}\left(\lambda_{2}\right)}{{n_{e}}^{-2}\left(\lambda_{2}\right)-{n_{o}}^{-2}\left(\lambda_{2}\right)}$$

Notice that *n*_e_(90°, *λ*_1_) = *n*_e_(*λ*_2_) = *N*_e_(*λ*_2_) in Figure 2, so Equation (7) can also be expressed by the following equation.

(8) $$\theta_{m}=\sin^{-1}\left(\sqrt{\frac{{n_{o}}^{-2}\left(\lambda_{1}\right)-{n_{o}}^{-2}\left(\lambda_{2}\right)}{{N_{e}}^{-2}\left(\lambda_{2}\right)-{n_{o}}^{-2}\left(\lambda_{2}\right)}}\right)$$

Thus, it can be seen that *θ*_m_ depends on the birefringence. In other words, it depends on the refractive index of the FW for ordinary light and the refractive index of the SHW for ordinary and extraordinary light. In the case of broadband light such as the femtosecond laser, *θ*_m_ is expected to change a lot because both *λ*_1_ and *λ*_2_ have wide wavelength bands.

Note that a focused collimated laser beam propagates ideally in the form of the Gaussian beam. Theoretically, the Gaussian beam converges and diverges from the beam waist, which is the focused area where the beam radius reaches the minimum value and is expressed by the following equation.

(9) $$w_{0}=\frac{2f\lambda_{1}}{\pi d}$$

The radius of beam waist by using a parabolic mirror depends on the focal length of the parabolic mirror denoted by *f*, the collimated beam diameter made incident to the parabolic mirror *d*, and the wavelength of FW *λ*_1_.

**Figure 3:**
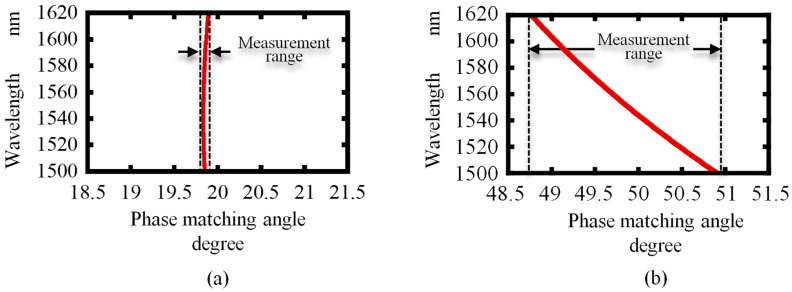
Wavelength-dependent phase-matching angle: (**a**) BBO; (**b**) MgO: LiNbO_3_.

Phase-matching angles for BBO and LiNbO_3_ are calculated using Equation (8) and the results are presented in Figure 3. By the calculation around the central fundamental wavelength of 1560 nm of an Er^3+^-doped fiber laser, the measurement range can be found and considered to select the suitable nonlinear optical crystal for the proposed angle measurement method.

As shown in the figure, the dispersion ranges of phase-matching angles for BBO, LiIO_3_, and LiNbO_3_ crystals within the fundamental wavelengths of 1500 nm to 1620 nm are 0.05°, 1.12°, and 2.17°, respectively. In the case of frequency-based measurement, the nonlinear crystal with a broader phase-matching dispersion range gives an advantage of a wide measurable range of angles. For the widest measurable range, LiNbO_3_ is the best suited to the proposed measurement system compared to the other crystals.

### 2.3. Effect of Crystal Diffraction on the Angular Displacement Sensitivity

**Figure 4:**
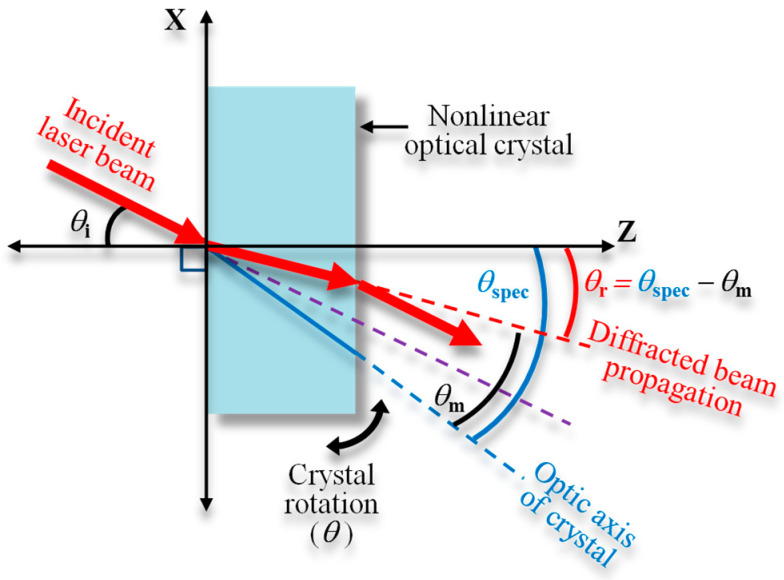
Schematic of refraction effect due to crystal diffraction; the change in direction of an incident laser beam through a nonlinear optical crystal.

The diffraction of a laser beam is one of the optical properties that cannot be eliminated in verifying the proposed method due to crystal usage. In general, the incident laser beam that passes through the boundary of two media with different refractive indices will propagate following Snell’s law, as schematically shown in Figure 4 and expressed as follows.

(10) $$\sin\theta_{i}=n_{c}\sin\theta_{r}$$

The figure above considers a case where the crystal is assumed to be a rectangular parallelepiped and the certain wavelength satisfies the phase-matching angle (*θ*_m_). The angle between the normal axis and optic axis crystal is marked as *θ*_spec_, the incident angle is *θ*_i_, the refraction angle is *θ*_r_, the refractive index of the crystal is *n*_c_, and the refractive index of air is assumed as 1. The relations of those parameters are described by the following equations.

(11) $$\theta_{r}=\theta_{\mathrm{spec}}-\theta_{m}$$

(12) $$\theta_{i}=\sin^{-1}\left(n_{c}\sin\theta_{r}\right)$$

It is noticed that crystal diffraction will affect the SHG calculation because the incident angle to the optic axis of the nonlinear crystal (*θ*) to achieve the phase matching condition must be greater than the assumed incident angle. Moreover, the sinc function in Equation (1) will also change. Thus, this condition should be taken into account in the calculation formula. The calculation result of both conditions, with and without the diffraction consideration, will be explained further in the next section.

## 3. Experiments and Results

**Figure 5:**
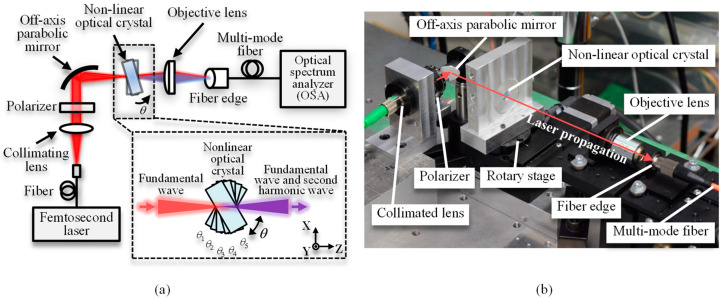
(**a**) Schematic of the experimental setup to observe FW and SHG phenomena using an off-axis parabolic mirror for beam focusing; (**b**) a photograph of the experimental setup.

The experiment was performed to confirm the feasibility of the proposed method. A schematic diagram and a photograph of the experimental setup are shown in Figure 5a,b, respectively. A commercial femtosecond laser (C Fiber, Menlo System, Munich, German) was employed as the beam source with the following specifications: a center wavelength of 1560 nm, an average output power of 15mW, a repetition rate of 100 MHz, and a pulse width of 150 fs. The beam was emitted from the fiber-connected femtosecond laser and traveled through the collimating lens (F280FC-1550, Thorlabs, Newton, NJ, USA), the polarizer (LPNIR050-C, Thorlabs), and the off-axis parabolic mirror (*f* = 50.8 mm, MPD029-G01, Thorlabs). The SHW of the laser light focused by a parabolic mirror was generated in the optical crystal. The nonlinear optical crystal was mounted on the rotary stage as a demonstration measurement target, and the SHW and unconverted FW were both injected into the multi-mode optical fiber with the objective lens and transmitted to the optical spectrum analyzer (AQ6370C, Yokogawa Electric, Tokyo, Japan), where the optical spectrum was recorded.

Firstly, a beam profiler (BP209-IR, Thorlabs) was installed at the focal point to investigate the laser focal spot size. To prevent damage to the beam profiler, another polarizer (LPNIR050-C, Thorlabs) was installed to reduce the intensity of light and to inject the laser beam into the beam profiler. The diameter of the focal spot was measured to be 34 µm in 1/e^2^ width.

**Figure 6:**
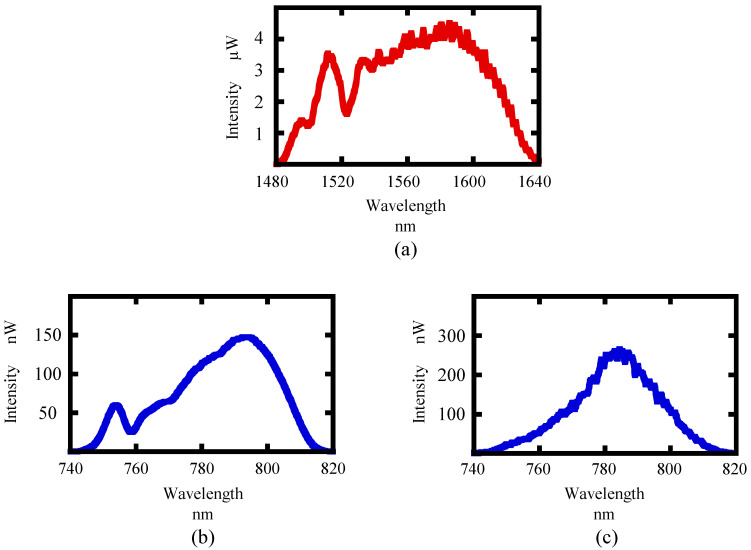
(**a**) FW spectrum before conversion; (**b**) second harmonic wave (SHW) spectrum focusing by a parabolic mirror; (**c**) SHW spectrum focusing by a lens.

In the next step of the experiment, the effect of the reduction of chromatic aberration by the parabolic mirror was confirmed. A beta-barium borate (BBO) crystal was employed as a nonlinear optical crystal, because the angular dispersion of BBO to the phase mismatch is rather small [25]. The characteristics of the BBO employed in this experiment are shown in Table 2. Figure 6a shows the light source spectrum before conversion. Figure 6b shows the SHW spectrum when the laser light was focused by a parabolic mirror. Figure 6c shows the SHW spectrum obtained by focusing the FW with a lens instead of a parabolic mirror. As can be seen in Figure 6b,c, the spectral trends of the two SHWs are quite different. In the case where the laser was focused by a parabolic mirror, the SHW spectrum had a shape that reflected the shape of the FW spectrum, in other words, a spectral peak around 750 nm was observed. In addition, the spectra are broader than the SHW spectra obtained when the lens was used to focus the light. On the other hand, the SHW spectrum with the lens focusing has a Gaussian-like shape. This is because when the light is focused by a parabolic mirror, the localization of SHWs due to chromatic aberration shown in [25] does not occur. As we discussed in [25], the conversion efficiency from the FW to SHW at the wavelength apart from the central wavelength decreases due to less intensity at the focusing point of the wavelength due to chromatic aberration. Therefore, by using a parabolic mirror, it is expected that a wide measurable range can be achieved by eliminating the effect of chromatic aberration.

**Figure 7:**
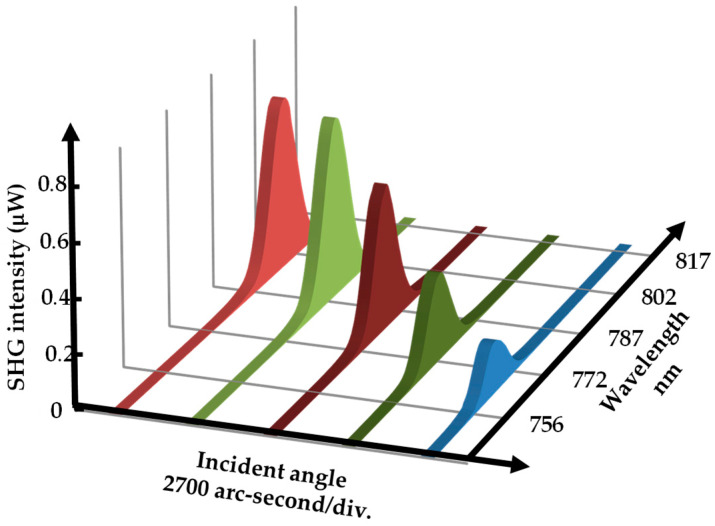
Characteristic of SHW spectra in different incident angles using LiNbO_3_ crystal.

Then, a feasibility study of the proposed angular measurement method based on second harmonic generation in the optical frequency domain was carried out. LiNbO_3_ was employed as a nonlinear optical crystal in the experiment. The properties of LiNbO_3_ used in this experiment are shown in Table 3. Figure 7 shows the picked experimental spectra observed when the target stage was rotated. The characteristics of the rotary stage are shown in Table 4. It can be seen that the spectral peak shifts with the rotational displacement of the rotary stage. Therefore, the central value of the spectra was evaluated using the following formula [42].

(13) $$\lambda_{C}=\frac{\sum_{i}\lambda_{i}I\left(\lambda_{i}\right)}{\sum_{i}I\left(\lambda_{i}\right)}$$

where *λ*_i_ is the *i*-th sampling wavelength and *I*(*λ*_i_) is the optical intensity at *λ*_i_. The cutoff strength is set to half of the maximum intensity as follows.

(14) $$I\left(\lambda_{i}\right)=\{\begin{array}{cc} 0 & \mathrm{if}\;I\left(\lambda_{i}\right)<0.5\max\left\{I\left(\lambda_{i}\right)\right\}\\ I\left(\lambda_{i}\right) & \mathrm{if}\;I\left(\lambda_{i}\right)\geq 0.5\max\left\{I\left(\lambda_{i}\right)\right\} \end{array}$$

**Figure 8:**
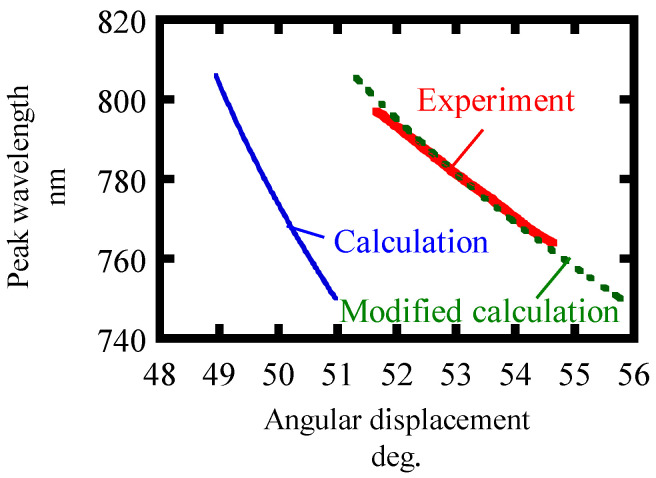
The sensitivity of the peak wavelength to the angular displacements.

The calculated *λ*_c_ dependence on the angular displacements is plotted in Figure 8. It is found to be in good agreement with the calculated values, which have been modified to account for refraction. The angular measurable range of over 10,000 arc-seconds was demonstrated.

**Figure 9:**
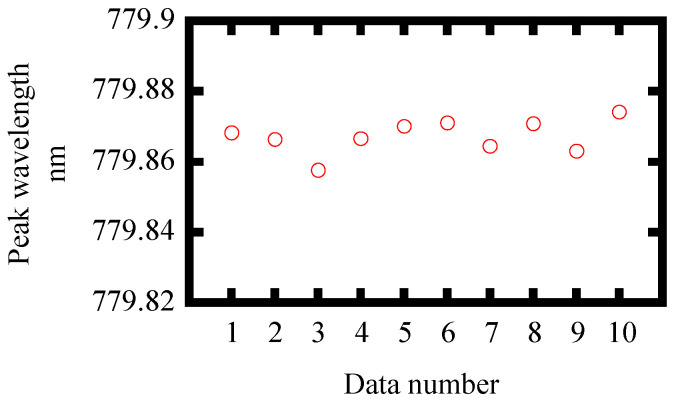
Experimental result of noise level using the standard deviation of the center-of-gravity wavelength calculation for each data point.

In order to obtain the resolution of the proposed method, we evaluated the noise level from experiments. Ten spectral measurements were made with a fixed rotary stage. The standard deviation of the center wavelength of spectra is shown in Table 5. Table 5 also shows the sensitivity of the proposed angular measurement method to angular displacements obtained from Figure 8 and Figure 9. In Figure 8, the slope of the modified calculation considering Snell’s law agrees well with the experimental results. Here, the experimental results are plotted in such a way that the angular displacement is consistent with the modified calculation at a wavelength of 780 nm. The resolution is defined by dividing the standard deviation of the noise by the sensitivity. A resolution of 3.00 arc-seconds was obtained. In addition, the dynamic range of the measurement method was also evaluated as a ratio of measurable range to resolution. Table 6 shows the characteristics of the angle measurement method in [25]. The angle measurable range is taken from 30% to 70% of the measurement response curve. The dynamic ranges of both measurements are comparable. Meanwhile, the advantage of the proposed method is that it has high measurement reproducibility because the observed wavelength is not affected by the intensity fluctuation of the FW which was unlikely in the previous research. By increasing the signal to noise ratio with higher incident FW power, an even higher resolution and dynamic range can be obtained. A supercontinuum source is another option to get a wider measurable range.

## 4. Conclusions

A new optical angle measurement method has been proposed by making use of the unique characteristics of high peak power and wide spectral range of the femtosecond laser pulses, which can generate second harmonic waves in a wide spectral range. For this purpose, we have proposed the method in the optical frequency domain. This method enables absolute angle measurement by the spectrum measurement of SHG. In the experiment, the validity of the use of a parabolic mirror has been demonstrated, where the chromatic aberration of the focusing beam has caused the localization of SHG in previous research. Besides, experimental results with the developed measurement system have demonstrated the feasibility of the proposed angular measurement. Moreover, it has been clarified that the refraction on the interface between the air and the nonlinear optical crystal should be taken into account. As a result, a measurable range of 10,752 arc-seconds and a measurement resolution of 3.00 arc-seconds have been achieved. The proposed method is expected to make a reliable measurement of tilt angle motions of a spindle of a machine tool as well as a reliable geometrical measurement, such as a surface profile measurement of a precision workpiece.

## Figures and Tables

**Table 1:** Medium-specific constants as the parameters for the empirical calculation of refractive indices [28,41].

Coefficients		Crystals	
		Beta-Barium Borate BBO	Magnesium-Oxide-Doped Lighium Niobate MgO: LiNbO_3_
A	For *n*_o_ (*θ =* 0°)	2.7359	4.8762
	For *N*_e_ (*θ =* 90°)	2.3753	4.5469
B (μm^−2^)	For *n*_o_ (*θ =* 0°)	0.001878	0.11554
	For *N*_e_ (*θ =* 90°)	0.01224	0.094779
C (μm^2^)	For *n*_o_ (*θ =* 0°)	0.01822	0.04674
	For *N*_e_ (*θ =* 90°)	0.01667	0.04439
D (μm^−2^)	For *n*_o_ (*θ =* 0°)	0.01354	0.033119
	For *N*_e_ (*θ =* 90°)	0.01516	0.026721

**Table 2:** BBO crystal characteristics.

Components	Value
Manufacturer	CASTECH Inc.
Name	BBO (*β*-BaB_2_O_4_)
Diameter	6.00 mm × 6.00 mm
Thickness	2 (±0.1) mm
Angle Tolerance	*θ* = 19.8° ± 0.25°; *φ* = 0° ± 0.25°
Flatness	≤*λ*/8 at 633 nm
Coating	S1: P-1560 nm; S2: P-780 nm

**Table 3:** LiNbO_3_ crystal characteristics.

Components	Value
Manufacturer	CASTECH Inc.
Name	5% MgO: LN
Diameter	5 (±0.1) mm × 5 (±0.1) mm
Thickness	2 (±0.1) mm
Angle Tolerance	*θ* = 47° ± 0.5°; *φ* = 30° ± 0.5°
Flatness	≤*λ*/6 at 633 nm
Coating	S1, S2: AR (1500–1600 nm/750–800 nm)

**Table 4:** Rotary stage characteristics.

Components	Value
Manufacturer	SURUGA SEIKI Co., Ltd.
Model number	KRB04017C
Travel Range	±8.5°
Resolution (pulse)	0.0067° (Full)
Repeatability Positioning Accuracy	±0.003°

**Table 5:** The experimental results of the proposed angle measurement system.

Standard Deviation 2 s (nm)	Sensitivity (nm/Arc-Second)	Resolution (Arc-Second)	Measurable Range (Arc-Second)	Dynamic Range
0.00943	0.00314	3.00	10,752	3584

**Table 6:** The characteristics of the angle measurement method in [24].

Resolution (Arc-Seconds)	Measurable Range (Arc-Seconds)	Dynamic Range
0.36	1040	2889
